# Reading Ability in Patients With Tuberous Sclerosis Complex: Results of Chinese Character Reading and Reading Comprehension Tests

**DOI:** 10.3389/fpsyg.2022.849334

**Published:** 2022-05-13

**Authors:** Hom-Yi Lee, Sheng-Hui Yang, Ji-Nan Sheu, Jeng-Dau Tsai

**Affiliations:** ^1^Department of Psychology, Chung Shan Medical University, Taichung, Taiwan; ^2^School of Medicine, Chung Shan Medical University, Taichung, Taiwan; ^3^Department of Paediatrics, Chung Shan Medical University Hospital, Taichung, Taiwan

**Keywords:** tuberous sclerosis, reading ability, neuropsychiatric disorders, reading tests, fluency test, comprehension test

## Abstract

**Background:**

Most tuberous sclerosis complex (TSC) patients have neurological disorders and are at high risk of academic difficulties. Among academic skills, reading ability is the most important academic skill. The study applied the Chinese character fluency test to measure the word recognition and reading comprehension of TSC children to observe whether they have the characteristics of reading disability, as an indicator of the spectrum of reading ability in TSC patients.

**Methods:**

The patients were assessed using the Chinese character fluency test and reading comprehension test to explore the differences in reading ability in terms of gender, age, epilepsy history, genotype, and intelligence level.

**Results:**

Of the 27 patients, the assessment of reading accuracy showed statistical differences between intellectual level > 80, PR (*p* = 0.024), and pass numbers (*p* = 0.018). For the fluency assessment, there was a difference between different intellectual level (*p* = 0.050). In the reading comprehension test, there was differences for intellectual level in positivity (*p* = 0.07) and pass numbers (*p* = 0.06).

**Conclusion:**

The Chinese character fluency and reading comprehension test measure the word recognition and reading comprehension and the spectrum of reading ability in TSC patients. All individuals with TSC, especially those with below average of intellectual ability, should be considered for potential academic difficulties.

## Introduction

In terms of pathophysiology in tuberous sclerosis complex (TSC), the disrupted brain connectivity causes the electrical activity responsible for transmitting signals involved in all neurological functions, including epilepsy and neuropsychiatric disorders (Tsai et al., 2021). In addition to intracranial lesions and epilepsy, the majority of individuals with TSC have a wide spectrum of neuropsychiatric disorders (Crino, 2016), including academic difficulties, such as reading, writing, mathematics, and spelling. About 30% of school-aged children with TSC have normal intellectual ability present with academic difficulties that require evaluation and support (de Vries et al., 2015). TSC patients with normal intellectual abilities are often not considered for individual education plans because of subtle academic difficulties. Given the apparent intellectual ability, TSCs are often interpreted as academic difficulties during school age, and educational systems do not consider and look for academic difficulties. For these reasons, the academic level needs to be considered as a distinct level of inquiry (Joinson et al., 2003).

Most TSC patients have neurological disorders and are at high risk of academic difficulties. Limited studies have applied questionnaires to investigate reading problems in school-age children with TSC. It was estimated 2%–20% of school-age children with TSC are reported to have academic difficulties, and the obstacles to academic skills are usually highly correlated with low self-esteem, social skills obstacles, and frustration (Prather and de Vries, 2004). Individuals of normal intelligence with TSC might be described by their parents as having academic difficulties in school performance. The TSC-associated neuropsychiatric disorders (TAND) checklist was developed (Bai et al., 2020), and the data collected through the checklist showed that 60.5% of TSC patients aged <18 years and 54.8% aged >18 years had certain degrees of academic difficulty (de Vries et al., 2018a). It can be seen that even if there is no systematic evaluation to confirm the relationship between TSC and academic difficulties.

Among academic skills, reading ability is the most important academic skill for up to 80% of TSC patients (Peters and Ansari, 2019). In this study, reading ability was used as an indicator to assess the academic level of TSC patients. Two standardized tests, namely the common Chinese character fluency test (Hung et al., 2012) and reading comprehension test (Ko and Chan, 2007) were used to assess whether the reading ability of TSC patients met the standards of reading disability. The study applied the Chinese character fluency test to measure the word recognition and reading comprehension of TSC children to measure whether they have the characteristics of reading disability, as an indicator of the spectrum of reading ability in TSC patients. This information might potentially provide a reference for family members and teachers when designing teaching plans for students with TSC.

## Materials and Methods

### Patient Selection and Study Design

The test subjects were all TSC patients at a single medical center. To identify reading ability, the required age was >8 years (elementary school grade 3). Individuals’ data, including gender, medical history, TSC gene, and intellectual level were recorded. To evaluate the degree of intellectual disability in the patients, an IQ test was conducted by a pediatric psychologist. Subsequently, the patients were assessed using the Chinese character fluency test and reading comprehension test to explore the differences in reading ability Written informed consent, with prior endorsement by all local institutional review boards, was obtained from all participants or their parents.

### Chinese Character Fluency Test

The Chinese character fluency test assesses the ability to recognize Chinese characters (Hung et al., 2012). Sixty common characters were included in the test. The participants were asked to read the character and make a word with the character within 1 min. The accuracy and fluency of character recognition were scored. Correctness refers to the number of correct character readings and word creations using the character. Internal reliability scores for each form at each age stage. Internal validity and construct validity were reported to be satisfactory. The cut-off point for a pass was defined as a 25 percent ranking (PR) to score the analysis difference, which identifies whether the subject may have a disability in reading accuracy and fluency.

### Reading Comprehension Test

The reading comprehension test is a measure of reading comprehension used for the screening of developmental dyslexia with norms established in Taiwan (Ko and Chan, 2007). Participants were asked to read a sentence or paragraph and then answer questions about the material. In addition to the items included for second graders (proposition combination, sentence comprehension, and paragraph comprehension), the text for other grades contained polysomic words, proposition combinations, sentence comprehension, and paragraph comprehension. All items were single-choice questions, and one point was given for each correct answer. The relationship of this test with the Chinese character reading test and listening comprehension measurements in Mandarin were all significant. For the reading comprehension test, the cut-off point PR was defined as 12, to identify reading comprehension ability.

### Statistical Methods

Non-parametric data were assessed by *t*-test, and the chi-square test or Fisher’s exact test was used to compare categorical variables. Statistical significance was set at *p* < 0.05. All statistical analyses were performed using SPSS for Windows (version 24.0; SPSS Inc., Chicago, IL, United States).

## Results

**Figure 1:**
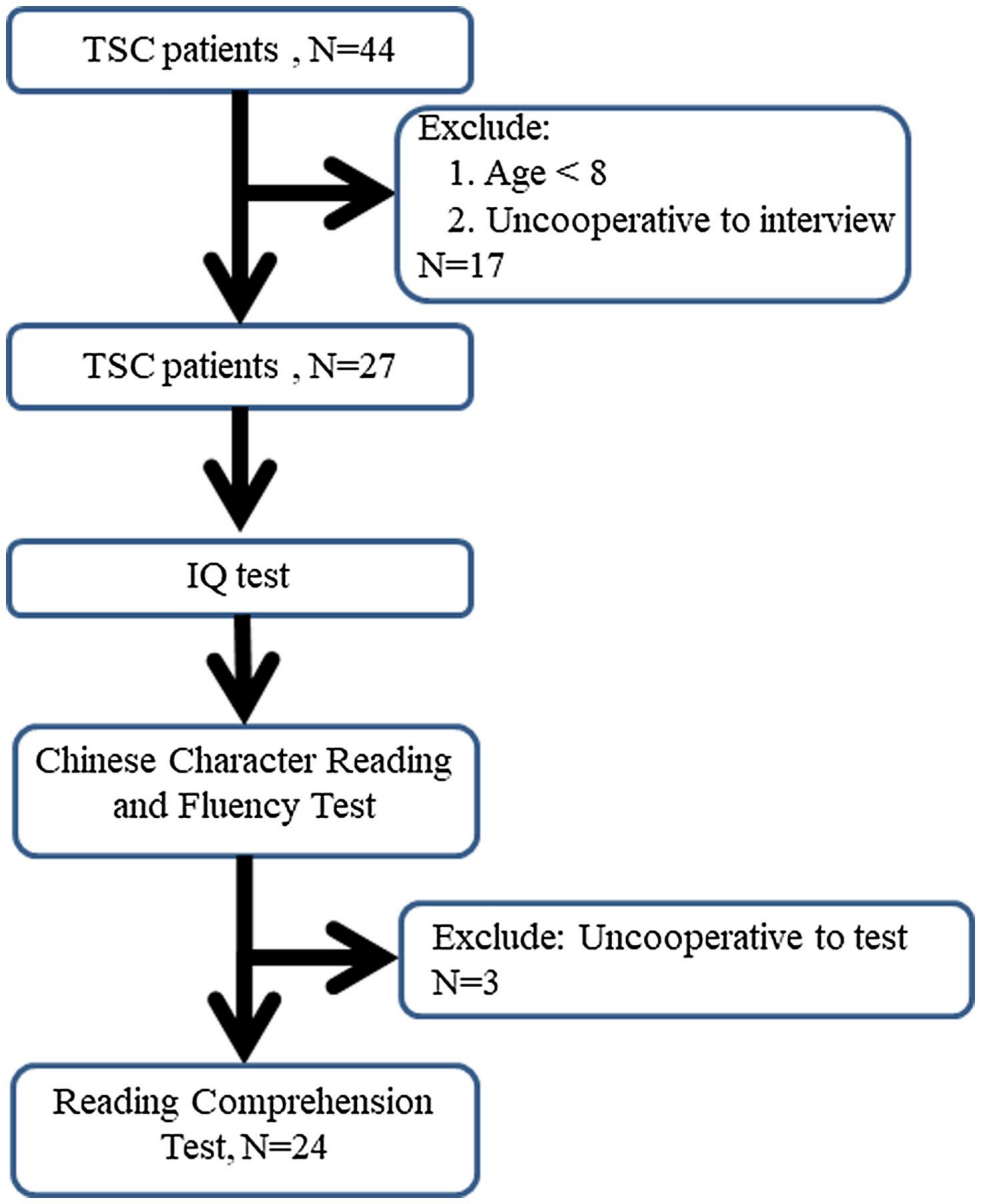
Twenty-seven tuberous sclerosis complex (TSC) patients have completed the Chinese Character Reading and Fluency Test and Reading Comprehension Screening Test.

Of the 44 TSC patients, 17 were excluded from the study due to poor compliance with the test and 27 participants completed the character fluency test and reading comprehension test (Figure 1). Of the 27 patients, nine were male and 18 were female; 10 patients were aged <18 years, and 17 patients were aged >18 years (Table 1). Fifteen (55.6%) patients had a history of epilepsy, and 12 (44.4%) were found to have normal intellectual levels. Of genotype, 15 (55.6%) were identified as TSC 2.

**Table 1:** Demographic data of TSC patients, *N* = 27.

Variable	Numbers	Percentage (%)
Gender		
Male	9	33.3
Female	18	66.7
Age		
<18	10	37.0
>18	17	63.0
History of epilepsy		
None	12	44.4
Positive	15	55.6
TSC gene		
TSC1	5	18.5
TSC2	15	55.6
NMI/ND	7	25.9
Intellectual level		
Normal (>80)	12	44.4
Borderline (70–80)	6	22.2
Disability (<70)	9	33.3

*TSC: tuberous sclerosis complex; Positive epilepsy history includes controlled and refractory epilepsy; NMI, no mutation identified and ND, not done*.

**Figure 2:**
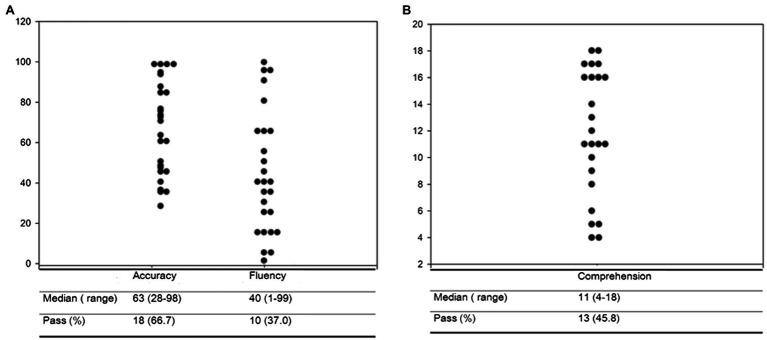
Chinese Character Reading test showed median PR 63 (range 28–98) and 18 (66.7%) pass in accuracy; In fluency, the median PR is 40 (range 1–99) and 10 (37.07%) pass in fluency test **(A)**. For Reading Comprehension Screening Test, the score showed median 11(4–18 range) and 13 (45.8%) pass the comprehension test **(B)**.

Overall results from the Chinese character reading test showed that the median PR was 63 (range 28–98), and 18 (66.7%) passed the accuracy test. For fluency, the median PR was 40 (range 1–99), and 10 (37.07%) passed the test. In the reading comprehension test, the median score was 11 (4–18 range), and 13 (45.8%) passed the test (Figure 2).

As shown in Table 2, the assessment of reading accuracy showed no statistical difference in terms of gender, age, epilepsy history, and TSC gene in PR or passing numbers. However, there were statistical differences between intellectual level > 80, PR (*p* = 0.024), and passing numbers (*p* = 0.018). For the fluency assessment, there was no statistical difference in PR in terms of gender, age, epilepsy history, TSC gene, and intellectual level. The percentage of pass numbers was not statistically different in terms of gender, age, epilepsy history, and TSC gene, but a difference was found in intellectual level (*p* = 0.050) (Table 3). As shown in Table 4, in the reading comprehension test, there was no statistical difference in positivity and pass numbers in terms of gender, age, epilepsy history, and TSC gene, but differences were found for intellectual level in positivity (*p* = 0.07) and pass numbers (*p* = 0.06).

**Table 2:** Accuracy of reading test in tuberous sclerosis complex, *N* = 27.

Parameter	Variable		*p*
Gender	Male (*n* = 9)	Female (*n* = 18)	
PR	70 (35–98)	66 (28–98)	0.675
Pass	6 (66.7)	12 (66.7)	0.661
Age	<18 (*n* = 10)	>18 (*n* = 17)	
PR	78.5 (45–72)	75 (28–98)	0.295
Pass (%)	6 (60.0)	12 (70.6)	0.439
Epilepsy history	None (*n* = 12)	Positive (*n* = 15)	
PR	66.5 (28–98)	66 (35–98)	0.889
Pass (%)	9 (75.0)	9 (60.0)	0.343
TSC gene	TSC 1 (*n* = 5)	TSC 2 (*N* = 15)	
PR	63 (45–98)	63 (28–98)	0.076
Pass (%)	4 (90.0)	10 (66.7)	0.517
Intellectual level	>80 (*n* = 12)	<80 (*n* = 15)	
PR	74 (47–98)	47 (28–94)	0.024
Pass (%)	11 (91.7)	7 (46.7)	0.018

*Data are presented as median (range) for PR and number (%) for pass. PR, percentile ranking and TSC, tuberous sclerosis complex; intellectual level is displayed in IQ test; significant is definite as *p* < 0.05*.

**Table 3:** Fluency of reading test in tuberous sclerosis complex, *N* = 27.

Parameter	Variable		*p*
Gender	Male (*n* = 9)	Female (*n* = 18)	
PR	35 (15–90)	47.5 (1–99)	0.456
Pass (%)	5 (55.6)	14 (77.8)	0.233
Age	<18 (*n* = 10)	>18 (*n* = 17)	
PR	30 (5–99)	50 (5–95)	0.288
Pass (%)	5 (50.0)	14 (82.4)	0.075
Epilepsy history	None (*n* = 12)	Positive (*n* = 15)	
PR	37.5 (5–99)	40 (1–95)	0.633
Pass (%)	8 (66.7)	11 (73.3)	0.706
TSC gene	TSC 1 (*n* = 5)	TSC 2 (*N* = 15)	
PR	45 (1–90)	35 (5–99)	0.833
Pass (%)	5 (100.0)	9(60.0)	0.091
Intellectual level	>80 (*n* = 12)	<80 (*n* = 15)	
PR	40 (15–99)	25 (1–65)	0.240
Pass (%)	16 (90.9)	5(55.6)	0.050

*Data are presented as median (range) for PR and numbers (%) for pass. PR, percentile ranking and TSC, tuberous sclerosis complex; intellectual level is displayed in IQ test; significant is definite as *p* < 0.05*.

**Table 4:** Reading comprehension test in TSC patients, *N* = 24.

Parameter	Variable		*p*
Gender	Male (*n* = 9)	Female (*n* = 15)	
Positivity	12 (5–18)	11 (4–18)	0.505
Pass (%)	5 (55.6)	7 (46.7)	0.673
Age	<18 (*n* = 9)	>18 (*n* = 15)	
Positivity	11 (5–17)	11 (4–18)	0.787
Pass (%)	5 (55.5)	7 (46.7)	0.673
Epilepsy history	None (*n* = 11)	Positive (*n* = 13)	
Positivity	14 (4–18)	11 (4–17)	0.825
Pass (%)	5 (45.5)	7 (53.8)	0.682
TSC gene	TSC 1 (*n* = 5)	TSC 2 (*n* = 12)	
Positivity	16 (9–18)	12 (4–17)	0.297
Pass (%)	3 (60.0)	6 (50.0)	0.563
Intellectual level	>80 (*n* = 11)	<80 (*n* = 13)	
Positivity	16 (4–18)	10 (4–17)	0.007
Pass (%)	9 (81.8)	3 (23.1)	0.006

*For positivity, data are presented as median (range) for positivity and number (%) for pass and TSC, tuberous sclerosis complex; intellectual level is displayed in IQ test; significant is definite as *p* < 0.05*.

**Figure 3:**
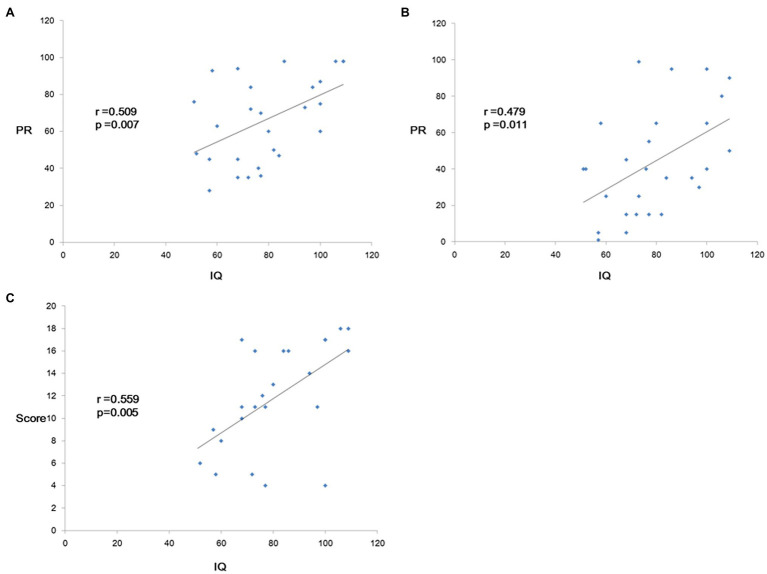
Linear regression between IQ score the Chinese Character Reading and Fluency Test and Reading Comprehension Screening Test. There are statistic significant of accuracy (*p* = 0.007, **A**) fluency (*p* = 0.011, **B**) and comprehension test (*p* = 0.005, **C**) when correlated with IQ test.

Linear regression between the character fluency test, the reading comprehension test, and IQ score was correlated. There were statistically significant differences in accuracy (*p* = 0.007), fluency (*p* = 0.011), and the comprehension test (*p* = 0.005) when correlated with the IQ test (Figure 3).

## Discussion

To date, current study is the first study to reveal the characters of reading ability in TSC population. The analysis results of the character fluency and comprehension tests are significantly associated with the different variable factors. Of all the variable factors, intellectual level significant impacts reading fluency performance, reading fluency pass rate, and reading theory correct answer rate.

The international TOSCA (TuberOus SClerosis registry to increase disease Awareness) study investigated over 2,000 TSC individuals. The study investigated difficulties in academic performance and the number of subjects who had been assessed with difficulties in academic skills. Up to 57.8% reported a lifetime history of academic difficulties (de Vries et al., 2018b). However, only 48.9% had received a formal assessment of these difficulties. Further research is required in this important spectrum of the TAND survey. The available results support the high rates of difficulties in academic skills and underline the lack of evaluation and necessary support in the educational system for such difficulties (Prather and de Vries, 2004). Thirty-six percent of school-age children of normal intellectual level with TSC were at high risk of academic difficulties in reading, writing, and mathematics (Kingswood et al., 2017), and up to 75% of students with TSC are in special education programs and receive services through the education system. In addition to specific learning needs, children with TSC are also at high risk for secondary deficits such as school refusal, anxiety about attending school, deficits in social skills, and low self-esteem (de Vries et al., 2018a). This indicates that all individuals with TSC, even those with normal intellectual ability, who may benefit from an individual education plan at school, should be evaluated for potential academic difficulties.

Reading difficulty is indicated when individuals have a disability in reading words or understanding what they read. It generally refers to difficulties in reading individual words and can lead to problems in understanding text. Reading difficulty involves phonological processing, reading fluency, speed, and reading comprehension. Most reading disorders result from specific differences in the way the brain processes written words and text (Hulme and Snowling, 2016). Individuals with reading disorders often have difficulties recognizing words they already know and understanding the text they read. They may also be poor spellers. Not everyone with a reading disorder displays noticeable symptoms. Early interventions and action programs for reading disability children often concentrate on promoting learning development. The clinical practice varies widely, and identifying the screening methods, diagnostic processes and support measures for which scientific evidence remain challenging (Swedish Council on Health Technology Assessment, 2014). In the current study, the performance of all subjects on the Chinese character fluency test is presented in a percentile ranking (PR), with a median PR of 63 (66.7% pass) in literacy accuracy, and an average PR of 40 (37.0% pass) in reading fluency. The comprehension tests of all the subjects showed an average PR of 11 (45.8% pass). This does not imply that character accuracy in most TSC patients is higher than the average level in the normal population; instead, it depends on the intelligence level of each individual. It has been shown that reading fluency is highly associated with cognitive ability, including rapid automatized naming, phonological awareness, and orthographic awareness (Leinenger, 2014). Under the above premise, intellectual level plays a more crucial role in reading ability than gender, age, epilepsy history, or genotype.

Impaired reading fluency has often been associated with impaired phonological awareness, even before the onset of reading instruction and connected texts composed of single words. It has also been associated with impaired naming speed for lists of stimuli and slowing in processing a series of stimuli, or slowing in the reading of a series of words that constitute a sentence (Leinenger, 2014). Using fMRI, it is compared the neural correlation of reading fluency in reading ability in the sentence reading paradigm that parametrically varied fluency demands by increasing the rate at which sentences were presented (Christodoulou et al., 2014). Impaired reading fluency was slower and less accurate across rates than typical readers, and subjects’ accuracy declined disproportionately as rates increased. Using diffusion tensor imaging, it was concluded that brain connectomes and organization were disrupted in TSC patients with different neuropsychological impairments (Tsai et al., 2021). The results revealed a trend toward decreased global integration and increased local segregation among TSC patients, which may indicate changes in brain networks, shifting toward structural regularity and leading to the impaired organization for efficient information transfer in these patients (Tsai et al., 2022).

Congenital or acquired factor may influence the reading ability in TSC patients. Of congenital factor, the TOSCA registry showed the genotype-intellectual phenotype correlation, concluded a higher frequency of intellectual and academic level were significant correlations with genotype, and academic difficulties were more common in individuals with TSC2 (de Vries et al., 2018a). However, it reported that 66.7% TSC1 and 42% TSC2 patients have normal intellectual ability, imply the genotype is not an absolute correlation factor with intellectual level. In current study, the children with TSC1 tend to outperform TSC2 in reading accuracy and reading fluency with border statistically significant. This is most likely related to the small number of cases in the study to make a statistical significance. Of acquired factor, children with reading difficulties might potentially cause by environmental factors, such as household income, low parental education, unstimulating home environment or inadequate instruction. When a school-age child is suspected of having significant reading difficulties, additional information about the educational, developmental, and family histories should be obtained (Hamilton and Glascoe, 2006). The caregivers should be asked to give details about individual’s academic performance in a range of skills.

This study descripts the reading ability of TSC population, focus on the characteristics of fluency and comprehension ability. However, there is not control population to view the difference between the TSC populations. The limited sample size in this study was due to the rarity of this disease, which may limit the generalizability of the results. As this is a cross-sectional study, the longitudinal follow-up of the reading tests could not be observed.

## Conclusion

The common Chinese character fluency test and reading comprehension test display the reading ability of TSC patients, measure the word recognition and reading comprehension of TSC children and the spectrum of reading ability in TSC patients. This information might potentially provide figures of reading ability. All individuals with TSC, especially those with below average of intellectual ability, should be considered for potential academic difficulties. The majority is likely to benefit from family members and teachers when designing teaching plans for students with TSC.

## Data Availability Statement

The original contributions presented in the study are included in the article/supplementary material, and further inquiries can be directed to the corresponding author.

## Ethics Statement

All procedures performed in studies involving human participants were in accordance with the ethical standards of Institutional review board of Chung Shan Medical University Hospital (CSMUH no. CS12245). Before participation, all the participants provided informed consent after the nature of the procedures had been fully explained.

## Author Contributions

H-YL conceived and designed the experiments. S-HY analyzed the data, prepared figures and tables and performed the experiments. J-NS contributed reagents, materials, and analysis tools. J-DT authored or reviewed drafts of the paper and approved the final draft. H-YL and S-HY contributed equally to this work. All authors contributed to the article and approved the submitted version.

## Funding

This study was supported by the grant (CSH-2021-A002) of Chung Shan Medical University Hospital, Taichung, Taiwan.

## Conflict of Interest

The authors declare that the research was conducted in the absence of any commercial or financial relationships that could be construed as a potential conflict of interest.

## Publisher’s Note

All claims expressed in this article are solely those of the authors and do not necessarily represent those of their affiliated organizations, or those of the publisher, the editors and the reviewers. Any product that may be evaluated in this article, or claim that may be made by its manufacturer, is not guaranteed or endorsed by the publisher.
